# Resistome Diversity and Dissemination of WHO Priority Antibiotic Resistant Pathogens in Lebanese Estuaries

**DOI:** 10.3390/antibiotics11030306

**Published:** 2022-02-24

**Authors:** Wadad Hobeika, Margaux Gaschet, Marie-Cécile Ploy, Elena Buelow, Dolla Karam Sarkis, Christophe Dagot

**Affiliations:** 1 Université de Limoges, INSERM, CHU Limoges, 87085 Limoges, France; wchobeika@gmail.com (W.H.); margaux.gaschet@unilim.fr (M.G.); marie-cecile.ploy@unilim.fr (M.-C.P.); 2 Microbiology Laboratory, School of Pharmacy, Saint-Joseph University, Beirut 17-5208, Lebanon; dolla.sarkis@usj.edu.lb; 3 Université Grenoble Alpes, CNRS, TIMC, 38000 Grenoble, France

**Keywords:** pathogens, resistome, estuaries, Lebanon

## Abstract

Anthropogenic pressure is known to be a key driver of antimicrobial resistance (AMR) dissemination in the environment. Especially in lower income countries, with poor infrastructure, the level of AMR dissemination is high. Therefore, we assessed the levels and diversity of antibiotic-resistant bacteria (ARB) and antibiotic resistance genes (ARGs) in Lebanese rivers at estuaries’ sites (*n* = 72) of the Mediterranean Sea in spring 2017 and winter 2018. Methods: A combined approach using culture techniques and high throughput qPCR were applied to identify ARB and ARGs in rivers along the Lebanese coast. Results: Multidrug-resistant Gram-negative (*Enterobacterales* and *Pseudomonas* spp.) and Gram-positive bacterial pathogens were isolated. Levels of ARGs were highest in the winter campaign and areas with high anthropogenic activities and population growth with an influx of refugees. Conclusion: Qualitative analysis of ARB and the analysis of the Lebanese estuaries’ resistome revealed critical levels of contamination with pathogenic bacteria and provided significant information about the spread of ARGs in anthropogenically impacted estuaries.

## 1. Introduction

Antimicrobial resistance (AMR) is a One Health crisis aggravated by the lack of water and pollution management on a global scale [1,2]. Anthropogenic activities are demonstrated to be the key drivers of AMR dissemination in the environment [3,4,5,6], subsequently altering its ecosystems [7,8,9]. Through discharge of treated or untreated wastewater (WW) effluents into surface water, a high abundance of antibiotic resistance genes (ARGs), antibiotic-resistant bacteria (ARB), and mobile genetic elements (MGEs) mixed with a cocktail of micropollutants and drug residues are continuously disseminated into the environment [10,11,12,13]. Additionally, agricultural practices such as soil fertilization with manure and sludge, or irrigation with WW effluents, further expand the environmental background levels of pollutants associated with the dissemination of antimicrobial resistance [9,14]. In Lebanon, rules to control the overuse and misuse of antibiotics for treatment, growth promotion, and prophylaxis in agriculture and animal husbandry are not strictly implemented [15,16], which would likely contribute to increasing AMR in the Lebanese and connected environments [17,18].

The surveillance of AMR in the environment is often assessed through ARG quantification [19,20]. High abundances of ARGs were found to be associated with fecal contamination [21,22,23].

Estuaries are transitional zones between rivers and sea bodies [24,25], exhibiting properties of marine and freshwater and underlining continental–oceanic interactions [26]. Estuaries are also considered as ecosystems that are broadly anthropized [24,27]. Contaminants can reside for prolonged periods in estuarian ecosystems due to the tidal streams [28,29]. The levels of ARB and ARGs and their spatial–temporal dissemination in Lebanese estuaries remains understudied [26]. The Mediterranean coastline is densely populated with a large anthropic footprint, i.e., intensive fishing, shipping, recreational, and industrial activities [30]. Our study aimed to assess the level of AMR dissemination in the Mediterranean Sea through the Lebanese estuaries. The objectives of this study were to monitor in the Lebanese river estuaries, the dissemination of i) ARB, notably extended-spectrum β-Lactamase-producing-*Enterobacterales* (ESBL-E), multidrug-resistant *Pseudomonas aeruginosa*, and methicillin-resistant *Staphylococcus aureus* (MRSA), and ii) the resistome through the detection of ARGs and MGEs.

## 2. Results

### 2.1. Bacterial Culture

In total, we isolated 19 different bacterial species in different quantities in spring and winter from the estuarine water samples along the Lebanon coastline (see the Materials and Methods section) (Table 1). From 1 mL of the water samples cultivated on selective media, we obtained 50 and 10,064 CFUs (colony forming units) resistant *Enterobacterales* and 41 and 43 CFUs resistant *Pseudomonas* spp. in spring and winter, respectively. However, among the 10,064 CFUs in winter, 10^4^ corresponded to the same bacterial species (*Hafnia alvei*) in the Beirut estuary. A more diverse panel of *Enterobacterales* species was isolated in spring. *Enterobacterales* and/or *Pseudomonas* spp. strains were isolated from all samples, except the Bared River. Moreover, *Enterobacterales* were not detected in the Kaleb River and *Pseudomonas* spp. were not found in the Zahnari river. MRSA isolates were detected only in spring. 

### 2.2. Susceptibility Profiles

We performed susceptibility testing for a panel of *Enterobacterales* (33) and *Pseudomonas* (39) strains. The results are shown in Table 2. We found 16 out of the 33 *Enterobacterales* and 6 out of the 39 *Pseudomonas* strains tested that expressed an ESBL phenotype detected with the double-disk synergy test according to the European Committee on Antimicrobial Susceptibility (EUCAST) v8 guidelines. 

Resistance towards the tested antibiotics was broadly disseminated. All tested strains of *Pseudomonas* in spring were susceptible to gentamicin. In winter, we detected two *Pseudomonas aeruginosa* strains with an ESBL synergy test, one in Janoubi and one in Beirut. We detected five strains resistant to imipenem in spring, three ESBL-positive *Pseudomonas luteola* strains (two in Aarqa and one in Beirut), and two *Enterobacterales* strains (one ESBL Klebsiella oxytoca in Aarqa and one Serratia marcescens in Qadicha). No resistant strains to imipenem were detected in winter.

### 2.3. Targeted Resistome Assessment by High-Throughput qPCR

**Figure 1:**
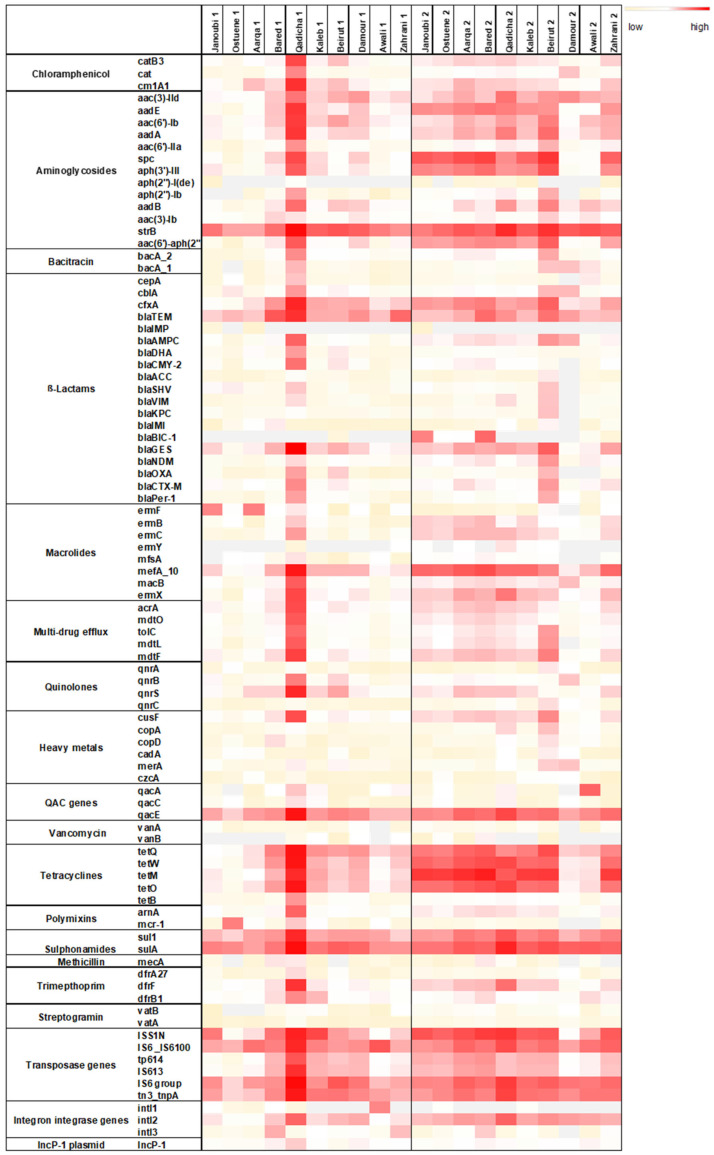
Heat map depicting the normalized abundances to the 16S rRNA gene of each targeted gene in spring (1) and in winter (2) in the 10 estuarine Lebanese river samples. Three-color legend with red: high normalized abundance, white: medium normalized abundance, and light yellow: low normalized abundance. Grey fields: gene undetectable. qac genes: genes conferring resistance to quaternary ammonium compounds.

Total DNA was extracted from triplicate river samples collected at the 12 estuaries of the major Lebanese rivers. The resistome analysis could only be performed on 10 rivers during both sampling campaigns since for 2 rivers DNA concentration was not sufficient. Figure 1 depicts the abundance of each targeted gene normalized to the 16S rRNA encoding gene. 

Overall, a higher normalized abundance of the targeted resistome was detected during the winter sampling campaign (Figure 1). Normalized abundance of individual ARGs was markedly high for the Qadicha estuarine during the spring sampling campaign, compared to all other estuaries.

The marker IncP-1 was detected in all river estuaries. IncP-1 plasmids are highly promiscuous and considered as anthropogenic markers in the environment. They frequently carry multiple ARGs and are suggested to be important vectors of ARGs and integrons [31].

Noticeably, transposase genes that are associated with the dissemination of ARGs amongst bacteria were detected in high normalized abundance in most river samples, especially during the winter sampling campaign. Individual resistance genes such as the *strB*, *spc* (conferring resistance to streptomycin, spectinomycin), the *mefA* gene (conferring resistance to macrolides), the tetracycline resistance gene *tetM*, and the resistance gene *sulA* conferring resistance to sulphonamides, were also detected in higher normalized abundance in different rivers during the winter sampling campaign.

**Figure 2:**
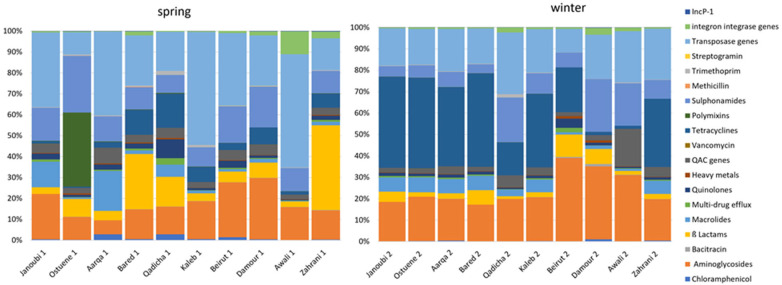
Proportional abundances of targeted genes grouped into gene families according to their function per river in spring and in winter in 10 estuarine Lebanese rivers samples. *qac* genes: genes conferring resistance to quaternary ammonium compounds.

Figure 2 exhibits different resistome signatures between the two sampling campaigns, with a higher diversity between individual rivers in spring than in winter. The resistome signature is less variable and remarkably similar between the northern sampled estuaries (Janoubi, Ostuene, Aarqa, and Bared) during the winter campaign.

**Figure 3:**
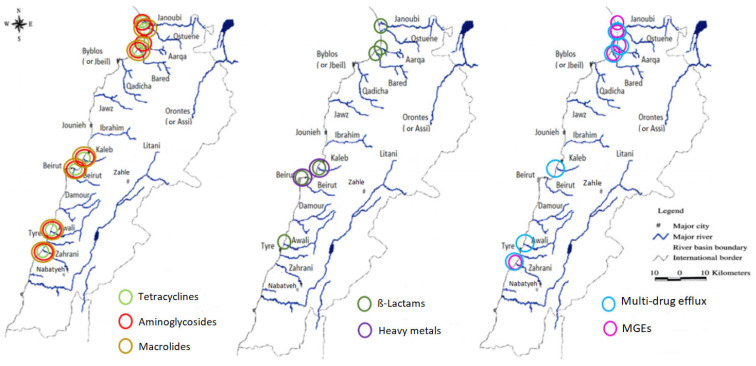
Graphical representation showing the estuaries where an increase in the mean normalized abundances of the ARGs families and MGEs (transposase genes, integron integrase genes, and the IncP-1 gene were grouped together as mobile genetic elements) from spring (2017) to winter (2018) was significant (*p* < 0.05).

Variation in normalized abundance for the different gene families in the estuaries during the two sampling campaigns in winter and spring was assessed using the non-parametric Wilcoxon’s test. Figure 3 depicts the estuaries where a significant difference in the mean normalized abundance was observed between spring and winter. The *p* values corresponding to all the significant variations are available in Supplementary Table S2.

Although we observed a significant increase for most ARG families in all samples from spring to winter, the mean normalized abundances of tetracyclines and β-Lactams, decreased from spring to winter in the Qadicha and Damour estuaries. In Qadicha, the aminoglycoside, macrolide, tetracycline, multidrug efflux, and transposase genes decreased significantly from spring to winter.

## 3. Discussion

Here we show that high levels of ARB and ARGs could be detected in all studied Lebanese rivers during all time points. Previous studies underlined the contamination of Lebanese rivers with fecal bacteria, especially *Escherichia coli* and coliforms, reaching 70.4% of the rivers in the North and 60% in the Bekaa region, which was above the acceptable threshold according to the French SEQ-EAU-2003 recommendations for irrigation water [32].

In our study, we showed that the levels of ARB and ARGs varied according to the location.

The Lebanese coastal belt from Tripoli to Tyre is an urbanized zone with significant agricultural activities [33,34]. The highest bacterial counts, (*Enterobacterales* (*Hafnia alvei* (10^4^)) in winter and *Pseudomonas luteola* (10^4^) in spring) were detected in the estuary of the capital Beirut. The highest normalized abundance of ARGs was observed in the Qadicha estuary in spring. The Qadicha river is a small river impacted by population growth, high industrial activities, and a chronic default of wastewater treatment. It has been shown that the estuary of this river was highly contaminated with pollutants [35], which may explain the high levels of ARGs and ARB detected here. The highest diversity of *Enterobacterales* was isolated from the Aarqa estuary in spring 2017, while a higher bacterial count was observed in winter (*Klebsiella pneumoniae* 50 CFU). The Aarqa estuary is located on the northern rural coast, inhabited by local and refugee communities with high agricultural activities.

**Figure 4:**
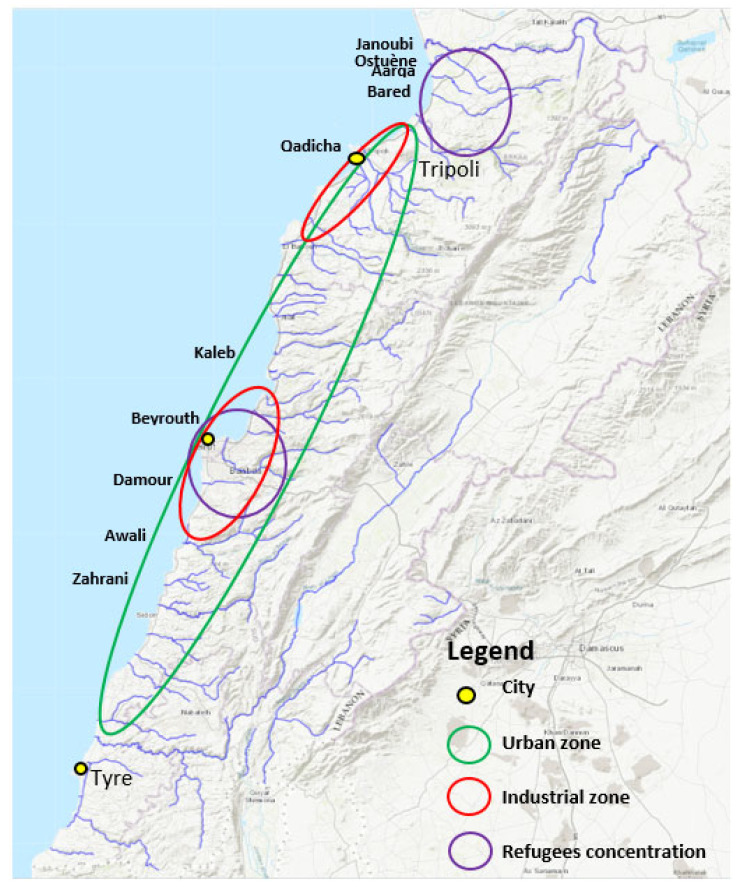
High anthropogenic impact locations. A map from “Rivers, Lebanon, 2012. https://maps.princeton.edu/catalog/stanford-wn533df2039” (accessed on 10 December 2021) representing the Lebanese rivers, modified to show locations on the Lebanese cost of the largest industrial zones (Beirut and Tripoli), the highest urbanized area, and the location of refugees, according to the UNHCR, UN-habitat 2014, and the UNRWA 2021 organizations.

Interestingly, we observed particularly high levels of ARGs in the North (Figure 3), which corresponds to the area with the highest number of inhabitants and with a high density of refugees’ camps (Figure 4). Lebanon hosts the largest number of refugees per capita worldwide. The Syrian crisis in the year 2011 has escalated river water pollution, particularly on the northern coast, following the migratory inflow of displaced persons searching for water points and settling to find refuge while infrastructure is lacking [36]. This inflow has contributed to sustaining extreme hot spots of water stress in urban areas in informal settlements (the Northern coast or in the Bekaa region), while water networks and governance are already insufficient [32]. In addition to the efforts from humanitarian organizations such as the UNHCR and the UNICEF to improve the sanitary of a fraction of the displaced people by the implementation of water and wastewater facilities, local initiatives to strengthen and/or rehabilitate existing infrastructure also exist and must be continued to alleviate the vulnerability of displaced communities and to reduce the water stress in the country [37,38]. 

River water levels in Lebanon increase during spring due to the rise of underground water and the snowmelt [39]. The difference in water levels between seasons may contribute to the observed lower normalized abundances of the targeted resistome in spring compared with the winter season (Figure 1). Notably, we observed a higher diversity of ARB and ARGs in the spring sampling campaign (Figure 2). The observed diversity for the targeted resistome and ARB in spring may also reflect an impact due to higher recreational activities in spring compared with winter.

Most bacteria that were isolated from the river estuaries were highly resistant to β Lactams, with 16/33 *Enterobacterales* strains and 6/39 *Pseudomonas* strains expressing an ESBL phenotype. The targeted resistome analysis of the river water samples detected a panel of ESBL-encoding genes, including a high abundance of ESBL-encoding genes that were specifically detected in the Mediterranean Sea [40,41], such as *bla_TEM_* and *bla_GES_* in most river samples during both seasons.

The high normalized abundance detected for transposase genes in the estuaries in both sampling campaigns might be indicative of an increased dissemination of ARGs among the bacteria in Lebanese estuaries. Transposons in rivers have been suggested to drive the dissemination of ARGs [42]. It was recently shown that transfer between resistance plasmids and bacterial chromosomes is mediated by insertion sequences, mainly those belonging to the IS6 family, IS26, and IS6100 [43]. The clinically relevant *IS6*-group and *IS6100* genes were detected in high abundance in the estuaries studied.

The high prevalence of the tetracycline resistance genes (*tetQ*, *tetW*, *tetM,* and *tetO*) might be the result of antibiotic selective pressure, considering that tetracyclines persist for a long time in the environment and are used frequently in human and veterinary medicine and aquacultural and agricultural practices [44,45,46].

Studies in lower income countries with lack of sanitation measures and operational wastewater treatment systems have shown high levels of ARB and ARGs that were significantly correlated with human gut bacteria and pathogens [23,47,48]. Inversely, resistant *Enterobacterales* in the urbanized San Francisco Bay were absent from near-shore sediments, suggesting that urbanized estuaries may not constitute a major human exposure hazard when secondary and tertiary treatment operations and control measures for all wastewaters that drain into the studied environment are implemented [49]. Altogether, the presence of ESBL and carbapenemase-producing pathogens and corresponding ARGs in the Lebanon estuaries raises concerns and warrants further attention [50].

The presence of MRSA in Aarqa, Ostuene, and Awali estuaries detected in spring should be closely monitored since studies have clearly shown that *Staphylococcus aureus* and MRSA persist in anthropized river samples, presenting a potential source of the dissemination and transmission of resistant bacteria [51,52,53].

Currently, wastewater treatment plants (WWTP) in Lebanon mainly use secondary systems, although their status of operationality is undetermined [36].

Here, we confirmed the estuaries of Lebanon as putative host-spots for the dissemination of ARGs and resistant bacteria into the Mediterranean Sea. Recreational waters such as rivers and beaches have gained increased attention as having a central role in the persistence, dissemination, and emergence of antibiotic resistance [54]. Specific surveillance systems should be put in place that trace urban movements, contact with recreational waters, pollution levels, and WW treatment levels and management to trace the actual dissemination of antimicrobial resistance through recreational waters [55]. 

## 4. Materials and Methods

Lebanon is located in the eastern Mediterranean area along a coastal length of 220 km [30] and is characterized by a short, cold, and wet winter season from January to March, with annual rainfall ranging between 850 mm and 1800 mm/year, and by a dry summer [56,57]. The surface waters in Lebanon are under increased pressure from anthropic activities, i.e., urban, industrial, and recreational. Agriculture in the coastal zone in Lebanon requires irrigation with surface and groundwater, causing the depletion of water resources while increasing pollution according to the Food and Agriculture organization report of the year 2016 [36].

Figure 4 marks anthropogenic impact on the different geographic locations of Lebanon by indicating important urban zones, industrial zones, and activity, as well as zones with high population densities due to the influx of refugees.

Fifteen Lebanese rivers spread through or at the extension of the Mount of Lebanon for a dozen of km eastward before discharging in small catchment areas leading to the Mediterranean Sea [58]. The rivers spread over the territory, making it a dense network of watercourses at a less than 10 km distance from each other and sharing similar basin characteristics [58]. To evaluate the seasonal impact on the presence and abundance of ARB and ARGs, sampling campaigns were performed at two different periods.

**Figure 5:**
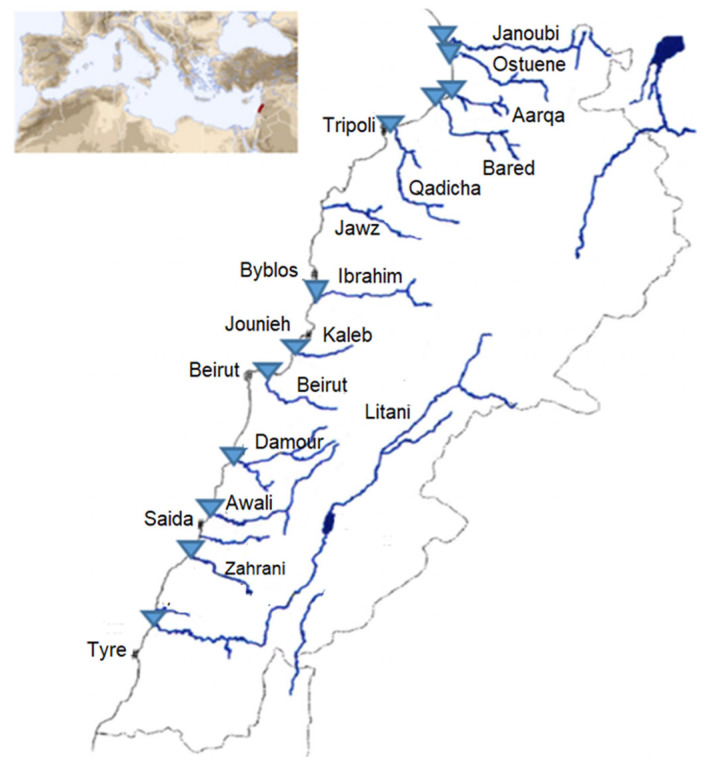
Sampling sites are represented as blue triangles on the map of the Lebanese rivers. Figure adapted from Houri and El Jeblawi (2007).

Among the rivers’ estuaries, twelve estuaries or mouths were sampled in triplicate in sterile cups in April 2017 and January 2018, resulting in *n* = 72 river water samples: 36 in spring and 36 in winter (3 samples of 60 mL each taken one after the other per river) (Figure 5). The samples from two rivers (Litani and Ibrahim) did not contain sufficient biomass and were excluded from the study. Weather conditions in terms of temperature with slight or no precipitation were similar, along with the month duration for each of the two sampling campaigns. Samples were transported on ice directly to our Lebanese laboratory for further analysis within 2 h. The exact coordinates registered for each sampling location as well as the temperatures are available in Supplementary Table S1.

The water samples were analyzed by the spread-plate method, culturing a V = 1 mL volume, using sterile rakes on MacConkey and Mannitol Salt agar selective of Gram-negative bacteria and *Staphylococcus* spp., respectively, with and without antibiotics. For Gram-negative enteric bacteria, we used ceftriaxone (2 mg/L), cefepime (4 mg/L), or ertapenem (0.5 mg/L). For *Staphylococcus aureus*, we used oxacillin (4 mg/L). All media were incubated at 37 °C for 48 h. For Gram-negative bacteria, species identification was performed with API^®^ 20 NE (for *Pseudomonas*) or API^®^ 20 E (for *Enterobacterales*) galleries (Biomérieux). For *S. aureus*, we used three phenotypic tests: catalase, DNAse, and coagulase.

The susceptibility testing was performed on Mueller–Hinton agar according to the European Committee on Antimicrobial Susceptibility (EUCAST) v8 guidelines. Antibiotic Bio-Rad^®^ discs used were: amoxicillin–clavulanic acid (20 µg–10 µg), cefepim 30 µg, ceftazidim (10 µg), aztreonam (30 µg), gentamicin (10 µg), amikacin (30 µg), piperacillin–tazobactam (30 µg–6 µg), and imipenem (10 µg) for Gram-negative bacteria, and fusidic acid (10 µg), cefoxitin (30 µg), trimethoprim–sulfamethoxazole (1.25–23.75 µg), gentamicin (10 µg), and ciprofloxacin (5 µg) for *S. aureus*.

For molecular analysis, the three water samples per river were filtered (total volume of water filtered = 180 mL), using a filtration ramp (Sartorius, Göttingen, Germany) on a sterile 47 mm diameter filter with a pore size of 0.45 µm (Sartorius, Göttingen, Germany). Microorganisms were recovered from filters and subject to DNA extraction for downstream analysis using the DNeasy PowerWater^®^ (Qiagen) adapted to water samples. All DNA samples were diluted or concentrated to a final concentration of 10 ng/µL for downstream qPCR and 16S rRNA analysis.

The Litani and Ibrahim rivers estuaries were excluded from the analysis, as the respective water samples contained insufficient biomass even after an additional 2 L water volume sampling and DNA extraction. 

We targeted 71 ARGs, 6 heavy metal resistance genes, and 3 genes encoding resistance to quaternary ammonium compounds, 9 MGEs (transposases *ISSW1*, *ISS1N*, *IS6100*, *IS613*, *IS6* group, *Tn3*, *ISCEc9*, *tp614*), and integron integrase genes (*intI1*, *intI2,* and *intI3*) [10]. The list of all the primers is published [10]. The 16S rRNA encoding genes were targeted to allow normalizing the abundance of individual resistance genes.

The targeted genes are grouped according to their function [10] with, in addition, the added environmental resistance marker: IncP-1 [31].

High throughput real-time PCR was performed using the Biomark microfluidic system from Fluidigm, in which every sample–gene combination is quantified using a 96.96 Dynamic Array™ IFCs (BMK-M-96.96, Fluidigm), as described previously [59]. A mean normalized abundance for each ARGs’ family and each estuary in spring and winter was calculated by dividing the cumulated abundance by the number of ARGs constituting the ARG family. The mean abundance of the ARGs families was then compared between spring and winter to assess a possible variation between the seasons. The estuaries where a significant variation was observed are listed in Supplementary Table S2.

## 5. Conclusions

To date, this is the first study to provide an accurate assessment and comparison of the targeted resistome in ten estuaries on the Lebanese coast. A combined approach using culture-based techniques and high throughput qPCR for the detection of ARB and ARGs identified Lebanese river estuaries as hotspots for antimicrobial resistance. This study highlights the need to implement regular antimicrobial resistance surveillance and improvement in wastewater management, in addition to the enforcement of regulations and guidelines stringency for sewage sludge or wastewater reuse, as per the new EU 2020/741 legislation of the European parliament.

## Figures and Tables

**Table 1:** Gram-negative bacterial species and Gram-positive MRSA isolated in the rivers’ estuaries.

River	*Enterobacterales*		*Pseudomonas* spp.		MRSA
	Species in Spring (CFU/mL)	Species in Winter (CFU/mL)	Species in Spring (CFU/mL)	Species in Winter (CFU/mL)	In Spring (CFU/mL)
Janoubi		*Escherichia coli* (7)		*Pseudomonas aeruginosa* (10) *Pseudomonas putida* (10) *Pseudomonas fluorescens* (5)	
Aarqa	*Enterobacter cloacae* (1) *Klebsiella pneumoniae* (5) *Hafnia alvei* (2) *Serratia marcescens* (1) *Serratia liquefaciens* (2) *Salmonella choleraesuis* (1) *Providencia rettgeri* (1) *Serratia rubidaea* (1) *Klebsiella oxytoca* (1) *Pantoea* spp. (16)	*Klebsiella pneumoniae* (50)	*Pseudomonas luteola* (14) *Pseudomonas fluorescens* (1) *Pseudomonas putida* (1)		(11)
Damour	*Escherichia coli* (2)		*Pseudomonas luteola* (1)		
Ostuene	*Enterobacter cloacae* (2) *Hafnia alvei* (2)		*Pseudomonas luteola* (7) *Pseudomonas putida* (1)		(1)
Zahrani	*Serratia liquefaciens* (3)				
Kaleb			*Pseudomonas luteola* (2) *Pseudomonas horizihabitans* (1)		
Bared				*Pseudomons putida* (3) *Pseudomonas fluorescens* (1)	
Beirut	*Enterobacter cancerogenus* (2) *Serratia marcescens* (1) *Klebsiella oxytoca* (1)	*Hafnia alvei* (10^4^)	*Pseudomonas luteola* (10^4^)	*Pseudomonas aeruginosa* (1)	
Qadicha	*Serratia marcescens* (2) *Hafnia alvei* (1) *Cedecea lepagei* (1)		*Pseudomonas luteola* (1)		
Awali		*Enterobacter amnigenus* (5) *Escherichia coli* (2)		*Pseudomonas fluorescens* (3) *Pseudomonas aeruginosa* (10)	(12)

**Table 2:** Resistance of the tested *Enterobacterales* and *Pseudomonas* species: numbers indicate the number of strains resistant out of the strains tested (%).

	*Enterobacterales*		*Pseudomonas*	
	Spring	Winter	Spring	Winter
Cefepime	16/27 (59%)	6/6 (100%)	6/31 (19%)	5/8 (63%)
Imipenem	2/27 (7%)	0/6 (0)	3/31 (10%)	0/8 (0)
Ceftazidime	17/27 (63%)	5/6 (83%)	6/31 (19%)	5/8 (63%)
Piperacillin–tazobactam	6/27 (22%)	4/6 (67%)	3/31 (10%)	4/8 (50%)
Gentamicin	5/27 (19%)	67 (17%)	0/31 (0)	1/8 (13%)
Amikacin	2/27 (7%)	1/6 (17%)	2/31 (6%)	1/8 (13%)
Aztreonam	21/27 (78%)	6/6 (100%)	27/31 (87%)	8/8 (100%)

## Data Availability

Not applicable.

## Supplementary Materials

The following are available online at https://www.mdpi.com/article/10.3390/antibiotics11030306/s1, Table S1: The exact coordinates of the sampling locations and the temperature recorded at the time of sampling, Table S2: Significant variations (*p* < 0.05) in ARGs families in the rivers between spring and winter.
