# Supplementary material for: Resistome Diversity and Dissemination of WHO Priority Antibiotic Resistant Pathogens in Lebanese Estuaries
Source: Antibiotics (Basel). 2022 Feb 24;11(3):306. doi: 10.3390/antibiotics11030306 (PMC8944630; doi:10.3390/antibiotics11030306)
Supplement: Supplementary file 1 [file antibiotics-11-00306-s001.zip › antibiotics-1534455-supplementary.pdf]

Supplementray Table S1. The exact coordinates of the sampling locations and the temperature recorded at the time of sampling

River	Coordonninates of the sampling locations	Temperatures in april 2017 in °C	Tempratures in january 2018 in °C
Janoubi Kabir	34° 38′ 04′′ N<br>35° 58′ 42′′ E	19	13
Ostuène	34° 36′ 07′′ N<br>35° 59′ 24′′ E	19	13
Aarqa	34° 32′ 58′′ N<br>35° 59′ 30′′ E	19	13
Al Bared	34° 30′ 31′′ N<br>35° 57′ 42′′ E	18	12
Qadicha	34° 26′ 48′′ N<br>35° 50′ 47′′ E	20	12
Ibrahim	34° 03′ 54′′ N<br>35° 38′ 35′′ E	20	13
Al Kaleb	33° 95′61′′ N<br>35° 58′ 80′′ E	19	13
Beyrouth	33° 54′ 06′′ N<br>35° 32′ 18′′ E	19	14
Damour	33° 42′ 17′′ N<br>35° 26′ 36′′ E	18	15
Al Awali	33° 35′ 20′′ N<br>35° 23′ 12′′ E	20	15
Zahrani	33° 29′ 33′′ N<br>35° 20′ 34′′ E	18	14
Litani	33° 95′61′′ N<br>35° 59′80′′ E	19	15

Supplementary Table S2. Significant variations (p < 0.05) of ARGs families in the rivers between spring and winter

	Significant increase (p value)	*Significant decrease* (p value)
Macrolides	Awali (p 0.028)	Qadicha (p 0.036)
	Bared (p 0.018)	
	Ostuene (p 0.043)	
	Beirut (p 0.018)	
	Kaleb (p 0.012)	
	Zahrani (p 0.018)	
Aminoglycosides	Awali (p *0.002)*	Qadicha (p 0.03)
	Bared (p *0.016)*	
	Ostuene (p *0.002)*	
	Beirut (p *0.002)*	
	Kaleb (p *0.001)*	
	Aarqa (p *0.01)*	
	Janoubi (p 0.023)	
	Zahrani (p *0.001)*	
Tetracyclines	Awali (p 0.043)	Qadicha (p 0.043)
	Bared (p 0.043)	Damour (p 0.043)
	Ostuene (p 0.043)	
	Beirut (p *0.043)*	
	Kaleb (p 0.043)	
	Janoubi (p 0.043)	
	Aarqa (p 0.043)	
	Zahrani (p 0.043)	
ß-Lactamase	Awali (p 0.03)	Qadicha (p 0)
	Bared (p 0.018)	Damour (p 0.025)
	Beirut (p *0.002)*	Zahrani (p 0.012)
	Kaleb (p 0.011)	
	Aarqa (p 0.02)	
	Janoubi (p 0.004)	
Heavy metals	Beirut (p 0.028)	
	Kaleb (p 0.046)	
MGEs	Aarqa (p 0.07)	Qadicha (p 0.017)
	Bared (p 0.021)	
	Ostuene (p 0.005)	
	Janoubi (p 0.021)	
	Zahrani (p 0.038)	
Multi-drug efflux	Awali (p 0.043)	Qadicha (p 0.043)
	Bared (p 0.043)	
	Ostuene (p 0.043)	
	Kaleb (p 0.043)	
	Aarqa (p 0.043)	
	Zahrani (p 0.043)	
